# Ductal Adenocarcinoma Ex Pleomorphic Adenoma of the Lacrimal Gland: a Rare and Morbid Malignancy

**DOI:** 10.1155/2020/1790106

**Published:** 2020-02-03

**Authors:** Prashanth Ashok Kumar, Shweta Paulraj, Kanish Mirchia, Seung Shin Hahn, Abirami Sivapiragasam

**Affiliations:** ^1^Department of Internal Medicine, SUNY Upstate Medical University, Syracuse, NY 13210, USA; ^2^Department of Pathology, SUNY Upstate Medical University, Syracuse, NY 13210, USA; ^3^Department of Radiation Oncology, SUNY Upstate Medical University, Syracuse, NY 13210, USA; ^4^Department of Hematology-Oncology, SUNY Upstate Medical University, Syracuse, NY 13210, USA

## Abstract

Carcinoma ex pleomorphic adenoma (Ca ex PA) is a rare malignant transformation of a benign primary pleomorphic adenoma (PA). We report the case of a 62-year-old male who presented with a swelling over his left temple. Imaging revealed a lytic lesion over the left orbital wall with soft tissue extension suggestive of malignancy. He underwent an en bloc resection of the mass with orbital exenteration, craniotomy, and reconstruction. Pathology demonstrated a lacrimal gland ductal adenocarcinoma arising from a PA which led to the diagnosis of ductal adenoCa ex PA. Postoperatively, he received chemotherapy with 6 cycles of cisplatin and concurrent radiation therapy (RT), but his course was complicated by recurrent bacterial meningitis and abscesses and he ultimately opted for comfort measures. Patients with PA of the lacrimal gland experience an insidiously enlarging painless swelling of the orbit with transformation to Ca ex PA highlighted by a rapid onset of bulbar enlargement, displacement, and often proptosis. Ductal adenoCa ex PA is aggressive with a poor prognosis and has no established standard of care. This case highlights the rarity of this condition and the need for more literature to help direct treatment.

## 1. Introduction

The lacrimal gland is an almond-shaped, bilobed, eccrine gland embedded in the superolateral aspect of the orbital wall and is composed of small lobules separated by connective tissue [1–4]. Lacrimal gland tumors occur in about 1 in 1,000,000 individuals per year and constitute 5-25% of all orbital malignancies [2, 3, 5]. Epithelial neoplasms are the most common followed by lymphoid and metastatic lesions. Epithelial lesions comprise around 50-60% of all benign and 40-50% of all malignant lesions [2]. Pleomorphic adenoma (PA) is the most common benign neoplasm of the lacrimal gland and has excellent prognosis after surgical excision like its salivary counterpart [3, 4]. It represents around 20% of all lacrimal gland tumors and 48% of all epithelial lacrimal gland lesions [2, 5]. Carcinoma ex pleomorphic adenoma (Ca ex PA) is a rare transformation of a benign primary PA to a malignant neoplasm [5, 6]. These rare tumors themselves show morphologic variations and one of them is ductal adenoCa ex PA [7, 8]. There are about 26 cases of ductal carcinomas of the lacrimal gland and very few cases of Ca ex PA, which makes our case a very uncommon presentation [5, 8]. The tumor is aggressive and carries a very poor prognosis. Most of the data on these tumors are extrapolated from its salivary gland counterpart. Even among salivary gland malignancies, Ca ex PA has a 5-year disease-specific survival rate of 37-44% which is lower than most other salivary gland tumors [6]. We present the clinical course of ductal adenoCa ex PA in a 62-year-old male and highlight the morbid nature of this malignancy.

## 2. Case Report

Our patient was a 62-year-old Caucasian male with a history significant for obstructive sleep apnea and osteoarthritis. He visited his primary care provider with a swelling over his left temple that was present for a long time but had gradually increased in size for the past several months. The swelling was around 3.5 × 3 cm in size on presentation with newly developing tenderness in the left temple. There was an initial suspicion for giant cell arteritis and hence a temporal artery biopsy was done which was negative. The swelling continued to progress causing proptosis, tearing, discharge from the left eye, and redness over the left temple and orbit. Initial laboratory investigations including SPEP, liver function testing, prostate-specific antigen, ESR, and CRP were within normal limits. CT maxillofacial with contrast showed a 3.8 × 2.7 cm lytic lesion on the left orbital wall with associated heterogeneous soft tissue that was suspicious for malignancy. CT head with and without contrast did not show any significant findings.

The interventional radiology (IR) team performed a CT-guided biopsy of the left periorbital soft tissue mass which showed soft tissue diffusely infiltrated by a moderately differentiated adenocarcinoma with a prominent cribriform pattern. Tumor cells had moderate cytologic atypia, numerous mitotic figures, and evidence of individual cell necrosis. Immunohistochemistry was positive for GATA-3 and negative for PSA and TTF-1. The consideration then was a primary adenocarcinoma of either lacrimal or sinonasal origin or a metastatic adenocarcinoma likely from the breast. MRI of the brain with and without contrast showed a fairly well demarcated, lobulated mass with mixed cystic and solid components along with heterogenous enhancement involving the left lacrimal gland and the lateral wall of the left orbit. The mass extended into the left suprazygomatic space and orbit. The left lateral rectus muscle was displaced. The overlying muscle in the left supra zygomatic masticator space demonstrated edema and enhancement. Nasal endoscopy did not reveal any evidence of malignancy in the upper aerodigestive tract. PET CT imaging from the skull to the thigh showed increased metabolic activity of the left orbital soft tissue mass with adjacent lytic changes involving the left orbit. There was no evidence of metabolically active lymphadenopathy in the head and neck nor any evidence of distant metastatic disease. ENT, neurosurgery, ophthalmology, medical oncology, and radiation oncology teams were involved in the care of the patient.

**Figure 1:**
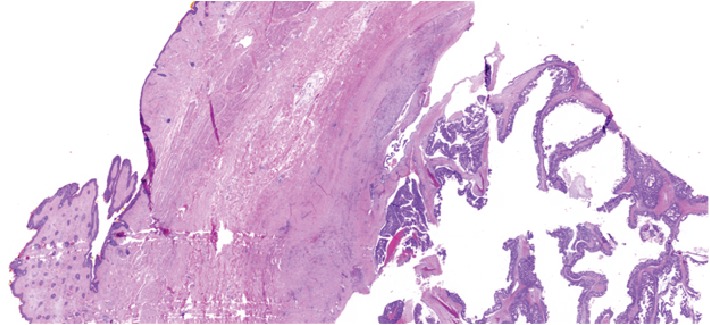
H&E sections (0.5x) showing relationship of the tumor with the overlying eyelid.

**Figure 2:**
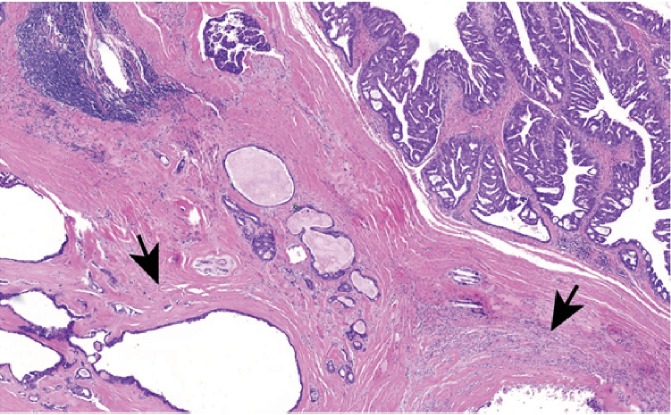
Hyalinized stroma of pleomorphic adenoma overtaken by the adenocarcinoma (H&E, 5x magnification).

**Figure 3:**
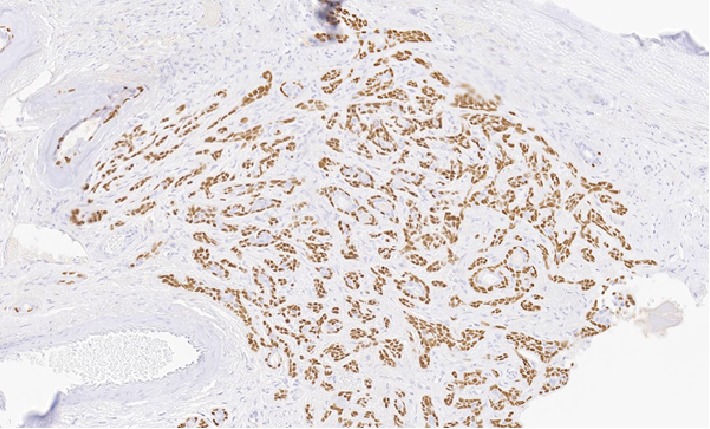
20x magnification of pleomorphic adenoma with positive p63 staining.

**Figure 4:**
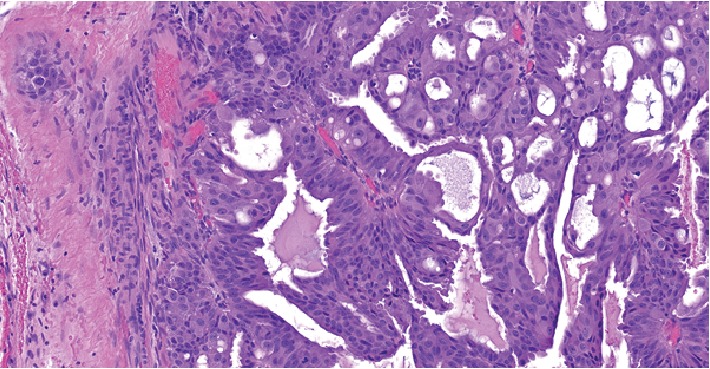
H&E sections (20x) showing neoplastic cells lining cystic structures with low- to high-grade pleomorphic nuclei.

**Figure 5:**
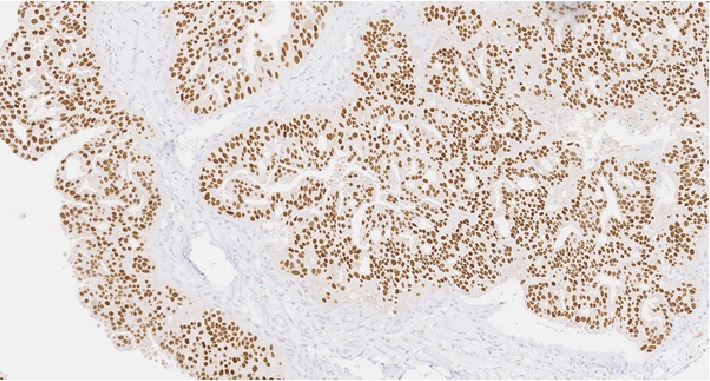
Carcinoma component showing positive androgen receptor (AR) staining (20x).

**Figure 6:**
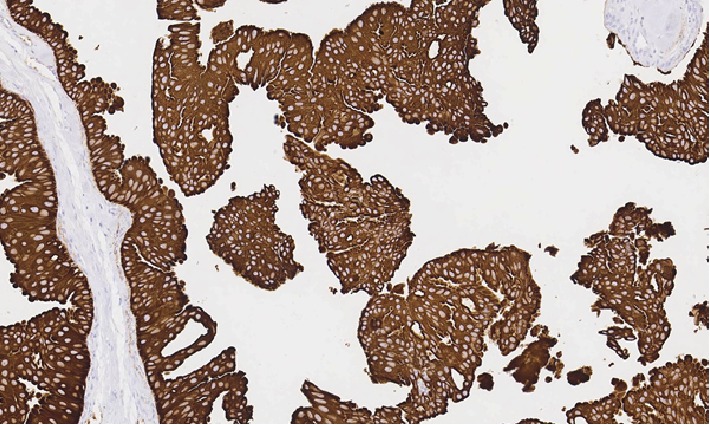
Carcinoma component showing positive staining for CAM 5.2 (20x).

**Figure 7:**
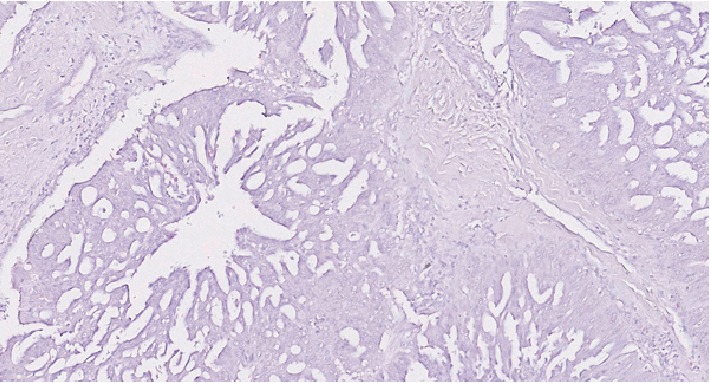
Carcinoma component showing negative staining for S100 (20x).

**Figure 8:**
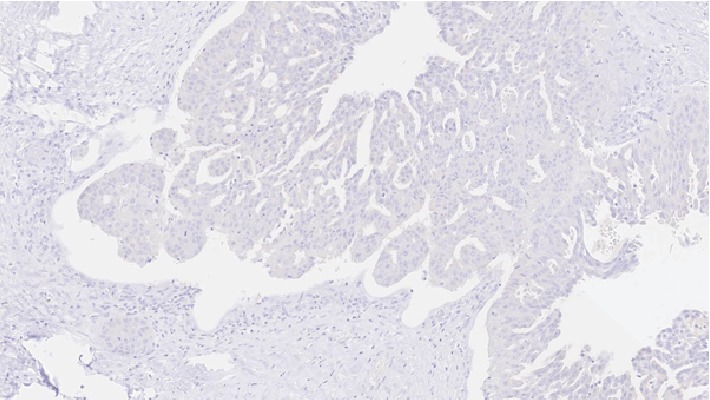
Carcinoma component showing negative staining for cytokeratin 5/6 (20x).

**Figure 9:**
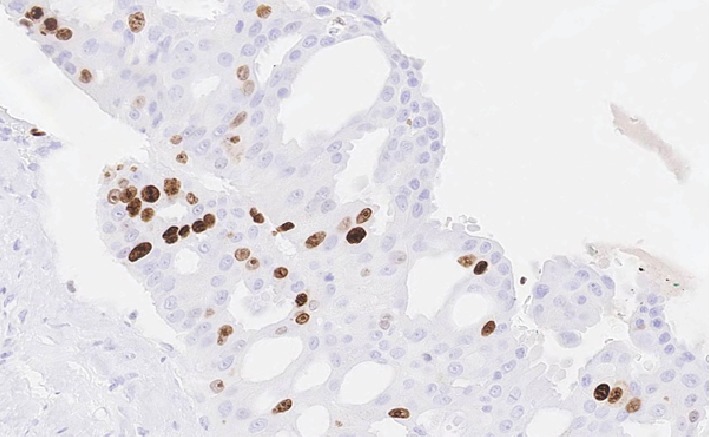
Areas of adenocarcinoma component showing focal areas of Ki 67 elevation (40x).

The patient underwent an en bloc resection of his lacrimal gland tumor which required orbital exenteration. Biopsies were sent. Since his tumor extended to the skull base, a craniotomy was performed and a small dural leak repaired with a pericranial graft.  Figure 1 shows the relationship of the tumor to the eyelid. Microscopic examination demonstrated the adenocarcinoma to be arising from a PA of the lacrimal gland (Figure 2), with the PA component demonstrating p63 positivity (Figure 3). The tumor showed an invasive cystic growth pattern with papillary and cribriform arch formations, invading the fibroadipose tissue and adjacent bone. The cystic structures were lined by neoplastic cells with apocrine features and nuclear pleomorphism ranging from low to high grade. Up to 8 mitotic figures/10 high-power fields were seen within the areas of high-grade features. Foci of invasive solid component were identified with cribriform architecture and high nuclear atypia (Figure 4). There was no evidence of perineural or lymphovascular invasion. Foci of hemorrhage, granulation tissue, and dystrophic calcification were also identified within the cystic component of the tumor. Immunohistochemistry showed the carcinoma cells to be positive for androgen receptor (AR), GCDFP15, HMWK903, and CAM5.2, while staining negative for S100, cytokeratin 5/6, p53, HER2/neu, progesterone (PR), and estrogen receptor (ER) (Figures 5–8). The Ki 67 index was focally elevated at 22% as shown in Figure 9. The overall findings supported the diagnosis of invasive ductal adenoCa ex PA of the lacrimal gland with the carcinoma component showing features ranging from low-grade papillary cystadenocarcinoma to intermediate-/high-grade ductal carcinoma.

Postoperatively, he received chemotherapy with 6 cycles of cisplatin with concurrent radiation therapy (RT). Six months after diagnosis and 2 months after completing chemoradiation, there was a suspicion for recurrence of the tumor and the patient had to undergo reexcision of the surgical site. Biopsies however did not show any definitive evidence of recurrence. Throughout his clinical course, he had multiple episodes of temporal abscesses and bacterial meningitis and had to undergo several craniotomies. Due to the complicated course and poor prognosis, the family decided to adopt comfort measures alone and the patient eventually succumbed to severe sepsis and cardiorespiratory arrest 1 year after his diagnosis.

## 3. Discussion

Lacrimal glands are sometimes considered a type of minor salivary gland and share histologic features with the main salivary glands [9]. Epithelial lacrimal gland tumors are grouped according to the classification of salivary gland tumors due to the common histopathologic features and lack of available data with regard to the former [10]. With PA, there always remains a risk of transformation into Ca ex PA and the risk increases with time [11]. There have been instances of malignant transformation noted 60 years after a diagnosis of PA [12]. Lacrimal and salivary PA and Ca ex PA have shown similar clinical and genomic profiles [13, 14]. Besides pure adenocarcinomas, there are other types of Ca ex PA such as myoepithelial carcinoma, adenoid cystic carcinoma, epithelial-myoepithelial carcinoma, squamous cell carcinoma, clear cell carcinoma, adenosquamous carcinoma, mucoepidermoid carcinoma, and acinic cell carcinoma. While adenocarcinoma is the most common type of Ca ex PA in salivary glands, there is insufficient information to comment on lacrimal Ca ex PA [15–18].

Patients with PA experience an insidiously enlarging painless swelling of the orbits over the years. Those with transformation to Ca ex PA experience a rapid onset of bulbar enlargement and displacement. Proptosis is often seen as in our patient [17, 19]. Lacrimal gland tumors are staged based on the TNM staging system and are assigned a grade based on the same [20]. Our patient would thus be T4cN0M0, G3.

The benign PA component is generally seen under the microscope as a pseudocapsule enclosed compilation of cuboidal ductal cells and polygonal or spindle-shaped myoepithelial cells in a chondromyxoid stroma [21]. The malignant component is characterized by nuclear pleomorphisms, atypical mitotic figures, hemorrhage, and necrosis. Various morphologies as mentioned above can be noticed [20]. Ca ex PA is noninvasive, minimally invasive, or invasive depending on its extension into the adjacent tissue. Noninvasive Ca ex PA remains within the fibrous capsule of the original PA. A minimally invasive tumor invades less than 1.5 mm in the extracapsular structures whereas an invasive Ca ex PA is characterized by greater than 1.5 mm invasion of the malignant component into the surrounding tissues [6, 22, 23]. Our case was thus invasive.

Hashimoto et al. showed that in salivary gland Ca ex PA, HER2 amplification played a significant role in the progression of the tumor and was associated with worse prognosis [24]. In Nambu and Tsukamoto et al.'s case of ductal adenoCa ex PA, HER2 gene amplification and protein overexpression was seen in the high-grade components, which was not the case in our patient. There is no available data on the use of anti-HER2-targeted therapies in ductal adenoCa ex PA of the lacrimal gland. AR positivity has been noted in some Ca ex PA, but no data on targeted therapies are available [8]. Ki-67 is a marker of DNA proliferation and malignant tumors usually have a high Ki-67 expression. The PA component being benign has a low expression, and the Ca ex PA is known to have a higher expression [6]. GCDFP-15 has been shown to be a biomarker for adenocarcinoma of the lacrimal gland, but its role in management is uncertain. Our patient was positive for the same [25]. p63 is a marker of myoepithelial elements and is positive in PA, whereas Ca ex PA is generally characterized by loss of p63 expression [26, 27]. Andreasen et al. in their report provide a summary of the demographics, stage, management, outcome, and certain receptor status of 25 cases of lacrimal gland ductal Ca from 1995 to 2016 [7].  Table 1 compares the present case to that of Nambu et al. who reported a high-grade invasive ductal Ca ex PA in 2017 [8].

Management of PA and Ca ex PA is surgical excision. If there is infiltration, the involved adjacent structures need to be removed followed by adjuvant RT and reconstructive procedures [18]. This is similar to our patient where orbital craniotomy was done. There is currently no standard protocol for chemotherapy or targeted therapy even for the more common salivary gland subtype. Sharon et al. reported a case of salivary HER2 positive Ca ex PA that was successfully treated with trastuzumab and capecitabine [22]. Similarly, in the salivary subtype, Chooback et al. showed a good response to a combination of cyclophosphamide, doxorubicin, and cisplatin. Paclitaxel-tamoxifen, cisplatin-5 fluorouracil, nedaplatin-docetaxel, and experimental wT1 peptide vaccine have demonstrated limited success in salivary Ca ex PA [22]. There is insufficient evidence of the successful use of chemotherapy or targeted therapies in lacrimal gland Ca ex PA in literature. When assessing the management of lacrimal Ca ex PA, it is to be noted that Ishida et al. and Daniel et al. used only surgical excision, whereas Nambu et al. used surgery with adjuvant RT [8, 18].

As seen with past experiences and in our patient, this rare tumor is aggressive and carries a very poor prognosis with a high predilection for recurrence and metastasis [20].

## 4. Conclusion

Ca ex PA is an extremely uncommon and aggressive tumor arising from a preexisting PA and carries a poor prognosis. Given the unpredictable nature of this tumor, we believe that it is important to make an early and correct pathologic diagnosis for which a high index of suspicion is needed. There are no guidelines to aid in management of this rare tumor. More case reports and, eventually, meta-analysis are needed to improve outcomes of this uncommon yet morbid malignancy.

## Figures and Tables

**Table 1:** Comparison of the present case and Ca ex PA reported by Nambu et al. in 2017 [8].

S. no		Present case	Nambu et al.
1	Age in years	62	72
2	Sex	Male	Male
3	Race	Caucasian	Japanese
4	Laterality	Left	Left
5	Presenting symptoms	Exophthalmos, redness, and swelling over the temple	Exophthalmos
6	Staging	T4cN0M0	T4cNxM0
7	AR	Positive	Positive
8	P53	Negative	Positive
9	HER2	Negative	Positive
10	GCDFP15, HMWK903, CAM5.2	Positive	Unknown
11	S100, cytokeratin 5/6	Negative	Unknown
12	ER, PR	Negative	Negative
13	Ki 67	Focally high-22%	Unknown
14	Treatment	Orbital exenteration and repair+chemotherapy+RT	Orbital exenteration and repair+RT
15	Outcome	Patient passed away	Unknown
